# Decontamination of SARS‐CoV‐2 contaminated N95 filtering facepiece respirators (FFRs) with moist heat generated by a multicooker

**DOI:** 10.1111/lam.13443

**Published:** 2020-12-31

**Authors:** Y.W. Choi, A.W. Richardson, M. Sunderman, M.J. Mladineo, P.H. Keyes, K.C. Hofacre, J.K. Middleton

**Affiliations:** ^1^ Battelle Memorial Institute Columbus OH USA

## Abstract

Decontamination of N95 filtering facepiece respirators (FFRs) is a crisis capacity strategy allowed when there are known shortages of FFRs. The application of moist heat is one decontamination method that has shown promise and is the approach approved in the Steris Steam Emergency Use Authorization (EUA). This effort examines the use of multicookers to apply moist heat, as they are available in retail stores and more affordable than methods requiring more sophisticated equipment. Four of five multicooker models examined met the acceptance criteria for the test and one model was selected for inactivation testing. Tests were performed on four different FFR models with SARS‐CoV‐2 suspended in culture media, simulated saliva or simulated lung fluid. Moist heat treatment reduced recoverable titres of SARS‐CoV‐2 virus to levels below the limit of detection in all tests. Furthermore, these four FFR models showed no loss in collection efficiency, inhalation resistance or visual damage after up to 10 decontamination cycles. Two (2) FFR models showed a slight change in strap elasticity (<9%). These data show that moist heat treatment using a multicooker is a viable option for FFR decontamination in a crisis capacity strategy.

## Graphical abstract

**Significance and Impact of the Study:** The potential to decontaminate N95 filtering facepiece respirators (FFRs) contaminated with SARS‐CoV‐2 in culture media, simulated saliva and simulated lung fluid using moist heat in a multicooker is demonstrated. Furthermore, the FFR models tested did not show degradation of performance when subjected to at most 10 decontamination cycles. This approach represents a low‐cost and simple FFR decontamination method that could be considered for use when shortages exist during an emergency, such as the ongoing COVID‐19 pandemic.

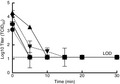

## Introduction

The potential for a pandemic to result in critical shortages of N95 filtering facepiece respirators (FFRs) has been recognized for over a decade (Anon. 2006). Since that time, research has been conducted on a variety of decontamination methods, including physical and chemical options, to extend the supply until additional supplies become available (Viscusi *et al*. 2009; Bergman *et al*. 2010; Fisher *et al*. 2011; Heimbuch *et al*. 2011; Viscusi *et al*. 2011; Lore *et al*. 2012; Heimbuch *et al*. 2014; Lin *et al*. 2018; Mills *et al*. 2018; Anderegg *et al*. 2020; Cadnum *et al*. 2020; Fischer *et al*. 2020; Grinshpun *et al*. 2020; Jatta *et al*. 2020; Li *et al*. 2020; Ma *et al*. 2020; Pauley *et al*. 2020; Schnell *et al*. 2020; Widmer and Richner 2020; Xiang *et al*. 2020; Zulauf *et al*. 2020). Moist heat is one of the methods that was shown to reduce the amount of infectious MS2 phage (Fisher *et al*. 2011; Li *et al*. 2020; Zulauf *et al*. 2020), methicillin‐resistant *Staphylococcus aureus* (MRSA) (Li *et al*. 2020), influenza virus (Heimbuch *et al*. 2011; Lore *et al*. 2012) and avian infectious bronchitis virus (Ma *et al*. 2020). Furthermore, other research on the stability of SARS‐CoV‐2 on surfaces and in aerosols has shown that the virus loses viability quicker at higher temperatures and relative humidity conditions (Aboubakr *et al*. 2020; Biryukov *et al*. 2020; Schuit *et al*. 2020), suggesting that this approach will be effective for decontamination of FFRs contaminated with SARS‐CoV‐2. It has also been shown that moist heat often does not degrade FFR performance for collection efficiency and inhalation resistance (Bergman *et al*. 2010; Fisher *et al*. 2011; Viscusi *et al*. 2011; Lore *et al*. 2012; Anderegg *et al*. 2020; Zulauf *et al.,* 2020), though some moist heat conditions have impacted FFR fit or the adhesion of the foam nose pad on certain FFR models (Bergman *et al*. 2010; Viscusi *et al*. 2011; Lore *et al*. 2012). Consistent with these studies, the U.S. Food and Drug Administration (FDA) issued an Emergency Use Authorization (EUA) for moist heat as described in the Steris Steam Decon Cycle in AMSCO Medium Steam Sterilizers (Hinton 2020), and 3M issued a technical bulletin stating that moist heat was an acceptable method for select FFR models (Anon. 2020). Both the EUA and 3M technical bulletin define moist heat as 65°C and 60–80% relative humidity for 30 min and indicate that moist heat can be applied for up to 10 cycles on select FFRs. It should be noted that moist heat does not include autoclave treatment, as this process has been shown to be detrimental to the FFRs (Grinshpun *et al*. 2020), as well as high temperatures in general, with 3M recommending that temperatures could be kept to 75°C or lower (Viscusi *et al*. 2009; Anon. 2020).

One benefit of using moist heat is that there are multiple options for generating the conditions that do not require specialized equipment, which may make this approach more accessible to a broader range of users (Fisher *et al*. 2011; Li *et al*. 2020; Zulauf *et al*. 2020). Such an approach may be necessary for FFR users who are not in the healthcare profession, as they are less likely to have access to more sophisticated equipment, are not covered by the EUA (Hinton 2020), and are expected to be a lower priority to receive replacement FFRs during a critical shortage. Researchers have used rice cookers, plastic containers, steam bags and convection ovens to generate moist heat, as just a few examples (Fisher *et al*. 2011; Anderegg *et al*. 2020; Li *et al*. 2020; Ma *et al*. 2020; Zulauf *et al*. 2020). The present research was conducted to identify a method of applying moist heat to FFRs that would not exceed the 75°C maximum temperature recommended by 3M and is affordable and accessible to users who are not covered by the EUAs currently in place. From a variety of options examined, multicookers with a sous vide function were identified. The relative humidity and temperatures achieved in different multicookers was measured, the efficacy of one multicooker in decontaminating FFRs inoculated with SARS‐CoV‐2 was assessed and the impact of the moist heat treatment on FFR performance was evaluated for up to 10 decontamination cycles. These results demonstrate that this method is a viable approach to accomplishing FFR decontamination by moist heat during periods of critical FFR shortages. The data herein may be used to request authorization for this method through official authorities.

## Results and discussion

### Evaluation of multicookers for the application of moist heat

**Figure 1:**
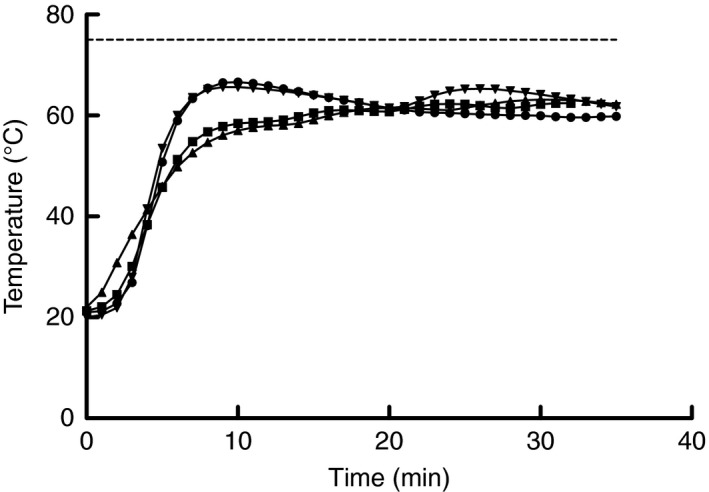
Temperature profiles for multicookers operated using the sous vide function with a set temperature of 65°C and set time of 30 min. Instant Pot (●), Gtime (■), Luby (▲), and GeekChef (▼).

Initial searches of ‘big box‐stores’ were conducted to identify devices that could be set to 65°C for 30 min, include moisture, be easy to use, require minimal to no modifications and have capacity to treat multiple masks simultaneously. Multicookers with the sous vide function were identified as the most promising option, and five models were obtained (see materials and methods below). The temperature and relative humidity in which FFRs would be exposed when treated was measured by the following steps: (1) each model had 500 mL of water placed in the pot; (2) a rack was placed into the pot to keep the sample from contacting the water; (3) a temperature/relative humidity data logger (Model UX100‐023A, Onset Computer Corp., Bourne, MA) was placed into a paper lunch bag (Great Value, Walmart, AK) and then set on the rack; (4) the lid was closed following multicooker manufacturer’s instructions; (5) the sous vide option was selected; (6) temperature was set to 65°C; (7) cook time was set to 30 min; and (8) the start button was pressed to begin the cycle. Note, the paper bag was used as a commonly available option to the use of the Steris Vis‐U‐All sterilization bags identified in the Steris Steam EUA. It was found that the inclusion of the paper bag was beneficial to the FFRs, as it prevented from absorbing too much water as reported by Fisher *et al*. (2011), while still allowing exposure to moisture during heating. One multicooker exceeded the 75°C temperature during the cycle, which is the maximum temperature recommended by 3M (Anon. 2020), and hence was not considered further in this study (data not shown). The recorded temperature profiles for the remaining four multicookers are shown in Fig. 1. Note, multicookers have a pre‐heating stage and the machine does not start the timer for the cycle until the set temperature has been reached. Average temperature and relative humidity inside the paper bag across all the multicookers was 61 ± 2·2°C and 94 ± 0·5% respectively. While similar to the conditions approved in the Steris Steam EUA and the 3M technical burden are similar to those measured, the differences were sufficient enough to recommend virus inactivation and performance degradation testing to confirm the efficacy of using multicookers for moist heat decontamination. The Instant Pot model was selected for subsequent testing for inactivation of SARS‐CoV‐2 on FFR material and degradation of performance following multiple decontamination cycles to conserve FFR quantities.

### Inactivation of SARS‐CoV‐2 on FFR coupons when subjected to moist heat

**Figure 2:**
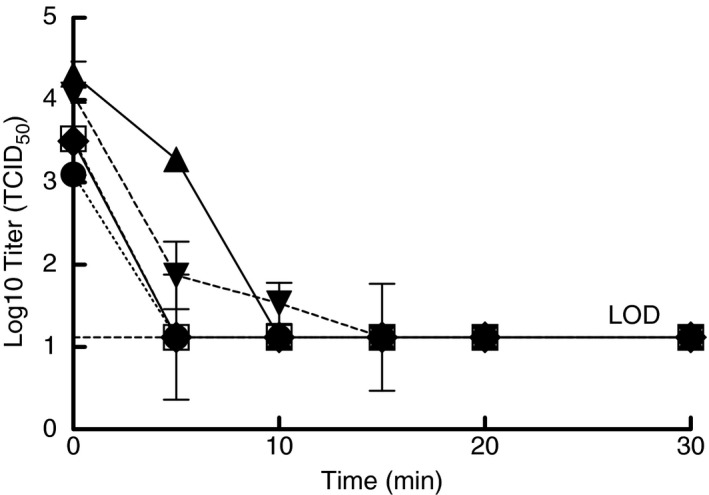
Inactivation of SARS‐CoV‐2 in culture media when spiked onto filtering facepiece respirator (FFR) coupons and treated with moist heat in a multicooker. Symbols are the mean of three samples and error bars are the standard deviation. 3M 1860 not in bag (●), 3M 8210 in bag (○), 3M 8210 no bag (♦), 3M 8511 in bag (▲), and NS 7210 not in bag (▼).

Due to the COVID‐19 pandemic and limited FFR supplies, SARS‐CoV‐2 inactivation tests were performed using coupons cut from whole FFRs (i.e. N95s) to conserve FFR quantities. During the first set of experiments, virus inactivation data were collected at six different timepoints over the total exposure duration of 30 min. Coupons from the 3M 1860 and NS 7210 FFR models were spiked with SARS‐CoV‐2 in culture media and subjected to moist heat in the multicooker with samples taken at the indicated times (Fig. 2). At the completion of this work, preliminary results from the performance degradation testing showed that the 3M 8210 FFR had reduced collection efficiency when exposed to moist heat for long durations (data not shown). Based upon the Steris Steam EUA and 3M technical bulletin, the moist heat protocol was updated to include placing the test samples in a paper bag to protect FFR performance. To confirm that the paper bag would not interfere with virus inactivation, 3M 8210 coupons spiked with SARS‐CoV‐2 in culture media were either subjected to moist heat directly or placed in a paper bag and then subjected to moist heat. Inactivation was the same for these samples, demonstrating that the paper bag did not prevent virus inactivation (Fig. 2). The results in Fig. 2 show inactivation of SARS‐CoV‐2 for 3M 1860 and NS 7210 coupons not placed in a bag and 3M 8210 and 3M 8511 coupons placed in a bag. Efficacy of moist heat for decontamination of coupons in paper bags from the 3M 1860 and NS 7210 FFRs was confirmed when testing was performed for SARS‐CoV‐2 in simulated saliva and simulated lung fluid. Though there were some differences between mask models in the early timepoints, SARS‐CoV‐2 virus was inactivated to levels below detection with moist heat by 20 min regardless of the FFR model, thereby demonstrating that an exposure achieved by setting the multicooker to a temperature of 65°C and time of 30 min with water present was sufficient to inactivate the virus to non‐detectable levels on each of the FFR models.

**Figure 3:**
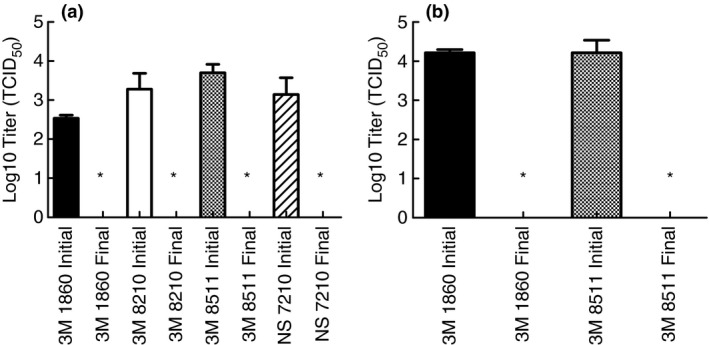
Inactivation of SARS‐CoV‐2 in simulated saliva (a) and simulated lung fluid (b) when spiked onto FFR coupons and treated with moist heat in a multicooker. Inactivation below detection (1·12 Log_10_ TCID_50_) indicated by an (*).

After demonstrating that moist heat treatment in the multicooker reduced infectious SARS‐CoV‐2 in culture media to levels below detection, subsequent testing was performed to verify the efficacy of the method when SARS‐CoV‐2 is located in body fluids that may actually contaminate an FFR. This step was necessary because previous research has shown that proteins in body fluids may provide protection for coronaviruses when subjected to decontamination processes (Rabenau *et al*. 2005; Darnell and Taylor 2006). For these tests, the stock virus was concentrated and resuspended in simulated saliva or simulated lung fluid (as indicated) prior to spiking onto FFR coupons. In the same manner as the previous tests, the spiked coupons were then placed into respective paper bags and subjected to moist heat generated by the multicooker set to 65°C with water for 30 min. The results in Fig. 3 show that moist heat was capable of inactivating SARS‐CoV‐2 in simulated saliva and simulated lung fluid to levels below detection.

### Inactivation of SARS‐CoV‐2 on nested FFRs when subjected to moist heat

**Figure 4:**
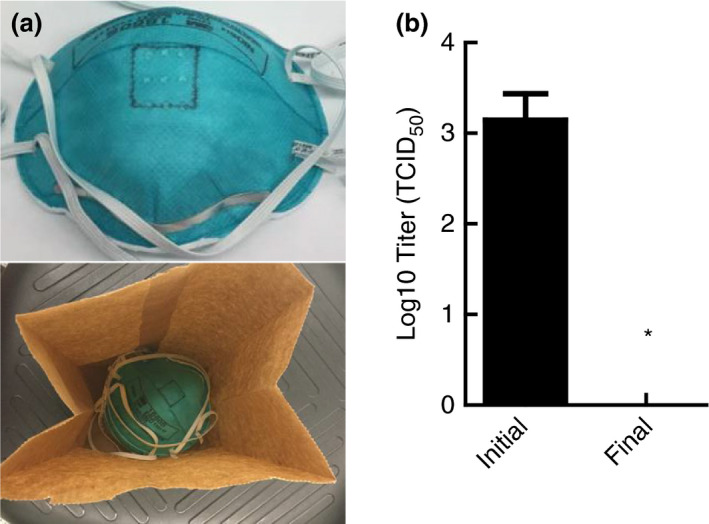
Inactivation of SARS‐CoV‐2 on nested 3M 1860 filtering facepiece respirators (FFRs). FFRs were marked with a 2 cm × 2 cm square where virus was inoculated before the masks were inoculated (a). Moist heat treatment in a multicooker inactivated the virus to levels below detection, even when nested (b). Inactivation below detection (1·12 Log_10_ TCID_50_) indicated by an (*).

The multicooker was tested to determine whether it could decontaminate multiple whole masks simultaneously. Due to the limitations in multicooker capacity, FFRs needed to be nested together (i.e. stacked atop each other) and placed within the same paper bag to increase throughput (Fig. 4a). Concerned that nesting of the FFRs might reduce the efficacy of moist heat, as demonstrated in coupon testing, three whole masks of 3M 1860 were spiked with virus in a dedicated 2 cm × 2 cm area, as shown in Fig. 4a, and permitted to dry. Afterwards, each FFR was nested atop each other and placed in a paper bag followed by treatment with moist heat in the multicooker. Upon completion of the decontamination treatment, the dedicated 2 cm × 2 cm sections were excised from each FFR, extracted and assayed for infectious virus. No viable virus was detected on any of the excised sections from the three FFRs as shown in Fig. 4b, thereby demonstrating that SARS‐CoV‐2 virus can be decontaminated when masks are nested.

### Assessment of FFR performance following multiple decontamination cycles

A successful decontamination method must not only reduce the amount of viable virus on the FFR, but it must do so without impacting the performance of the FFR. Early experiments were conducted to examine the impact of moist heat on FFR performance, including exposure to moist heat for 4 h at 52°C as shorter durations did not inactivate SARS‐CoV‐2 to levels below detection. It was found that FFR 3M 8210 absorbed over 10 g of water and some units had collection efficiencies below 95%. Excessive water uptake by some FFR models was also noted by Fisher *et al*. (2011). It was at this time that the updated guidance from 3M and the Steris Steam EUA was issued where sterilization bags were used (Anon. 2020; Hinton 2020). Paper bags were chosen as a commonly available substitute for sterilization bags and samples were tested again. It was found that FFR 3M 8210, as well as the other three models, absorbed <1 g of water when in a paper bag. Additionally, the collection efficiency of all four FFR models still exceeded 95% after moist heat treatment in a paper bag. Thus, the moist heat protocol was updated to use paper bags and all further testing of decontamination cycles for performance degradation were performed with the FFRs in paper bags.

Having determined that moist heat in the multicooker will inactivate virus to levels below detection, the impact of moist heat on FFR performance was assessed. Each FFR model was subjected to five cycles of moist heat decontamination as previously described with the FFR being allowed to dry for at least 30 min between each cycle. Upon completion of the fifth cycle, performance of the FFRs was tested by performing a visual inspection, determining the collection efficiency and inhalation resistance, and determining the change in strap elasticity (Table 1). All masks met performance criteria for collection efficiency (>95%) and inhalation resistance (<35 mmH_2_O) (Anon. 2019). There are no criteria for strap elasticity, which was measured as an indicator of fit, but the 3M 8210 and NS 7210 showed no change (p > 0·05 by one‐way analysis of variance [ANOVA]), while the 3M 1860 and 3M 8511 showed a small change (<10%) that is not expected to have a major impact on fit. Additional decontamination cycles were not performed for the 3M 1860 and 3M 8210 to conserve mask quantities because the Steris EUA and 3M technical bulletin already indicate that these masks can be subjected to 10 decontamination cycles. However, there is no guidance on how many moist heat decontamination cycles are acceptable for the 3M 8511 and NS 7210 FFRs. As a result, these models were subjected to 10 decontamination cycles and their performance evaluated. As shown in Table 1, all of these FFRs had performance that meets the specifications for collection efficiency and airflow resistance, no visual defects and no significant differences in strap elasticity. These results suggest that these two FFRs can be subjected to 10 moist heat decontamination cycles in a multicooker and maintain their performance.

**Table 1:** Summary of performance testing results for FFRs exposed to moist heat decontamination cycles

FFR	Cycles Completed	Collection Efficiency (%)	Inhalation Resistance (mmH_2_O)	Δ Strap Stress (%)	Δ Strap Max Load (%)	Visual Inspection Findings
3M 1860	0	99·7 ± 0·12	8·4 ± 0·6	N/A	N/A	N/A
	5	98·5 ± 0·6	6·6 ± 0·1	9	9	NVD
3M 8210	0	99·7 ± 0·37	7·0 ± 0·5	N/A	N/A	N/A
	5	99·2 ± 0·4	6·0 ± 0·1	NSD	NSD	NVD
3M 8511	0	98·9 ± 0·8	6·2 ± 0·4	N/A	N/A	N/A
	5	98·6 ± 0·3	5·9 ± 0·2	NSD	7	NVD
	10	98·7 ± 0·6	6·1 ± 0·2	NSD	NSD	NVD
NS 7210	0	99·5 ± 0·2	9·1 ± 0·3	N/A	N/A	N/A
	5	99·5 ± 0·2	8·4 ± 0·9	NSD	NSD	NVD
	10	99·6 ± 0·1	8·9 ± 0·8	NSD	NSD	NVD

Requirements for N95 FFRs are that the collection efficiency be greater than 95% and the inhalation resistance be less than 35 mm H2O. There are no requirements associated with strap elasticity and the percent change is shown as an indicator of fit. Visual inspection examined integrity of the filter media, nose pads, exhalation valve (if present), and strap attachments.

N/A, not applicable; NVD, no visual defects observed; NSD, no statistical difference.

## Materials and methods

### Cells

Vero (African green monkey kidney) clone E6 cells (ATCC Cat. No. NR‐596, Manassas, VA) were used to propagate stock SARS‐CoV‐2 and perform virus infectivity assays. Cells were incubated at 37°C with 5% carbon dioxide (CO_2_) in complete cell culture media (Dulbecco’s Modified Eagle Medium, Corning Cat. No. 10‐010‐CV, Corning, NY) supplemented with 2% foetal bovine serum (FBS) (Gibco Cat. No. 10082147, Carlsbad, CA) and penicillin‐streptomycin (Gibco Cat. No. 15140122).

### Virus

SARS‐CoV‐2 strain USA‐WA1/2020 was obtained from BEI Resources (Manassas, VA) and propagated in Vero E6 cells. All work performed with SARS‐CoV‐2 was done at Biosafety Level 3 and approved by Battelle’s Institutional Biosafety Committee. Following the 1‐hour binding period in Vero E6 cells with the virus seed stock, the virus lysate was harvested by scraping any remaining cells off the substrate after a 48‐ to 72‐h incubation time. The suspension was vortexed with sterile glass beads to break apart intact cells and then centrifuged at 800***g*** to remove cellular debris. The resulting supernatant was then aliquoted and frozen at −80°C in single‐use vials for testing.

The concentration of infectious virus was determined by a cell‐based assay in 96‐well plates to determine the median tissue culture infectious dose (TCID_50_). This assay was performed by serially diluting the virus stock suspension and transferring each of the dilutions to corresponding wells that contained Vero E6 cells. Determination for cytopathic effects (CPE) on the Vero E6 cell monolayer was performed after 72 hours of incubation. Quantitation of CPE was determined by using the Spearman–Karber method. The limit for detection of this assay was 13·1 TCID_50_ (Log_10_ TCID_50_ = 1·12).

### Filtering facepiece respirators

Four N95 FFRs were selected for this study. These FFRs were selected based upon their common use in the field, presence of unique features and availability given that FFRs were in extremely short supply during the conducting of this work. The FFRs used were 3M Models 1860, 8210 and 8511 (St. Paul, MN) and Northern Safety (NS) Model 7210 (Utica, NY). The 3M 1860 and 3M 8210 are commonly used by healthcare professionals, first responders and security professionals who interact with the public. The 3M 8511 is unique in this study since it has an exhalation valve, and the NS 7210 is from a different manufacturer to help understand whether the results generated may be applicable to multiple manufacturers.

### Multicookers

Multicookers with the sous vide function that could be readily obtained from retail stores were identified. The models purchased included the Instant Pot® Duo Evo Plus (Kanata, ON, Canda); Gtime Electric Pressure Cooker (Model GT‐60JT3, Amazon, Seattle, WA); Sous V Precision Cooker (Model: BSV‐A601, Amazon, Seattle, WA); GeekChef® 11 1 Multi‐Functional Cooker (Model YBW60P, Fairfield, NJ); and Luby Electric Pressure Cooker (Model GT606, Amazon, Seattle, WA).

Moist heat was applied to samples by placing 500 ml of water in the pot of each 6‐quart multicooker and then selecting the sous vide function with the temperature set at 65°C and timer set for 30 min per the user’s manual instructions for the specific multicooker. Samples were placed on a rack to separate them from the water. If the rack supplied with the system was not tall enough, then binder clips were attached to the feet to make the rack surface taller. For experiments using SARS‐CoV‐2, a custom rack was made to make manipulation of the samples in the Class III biosafety cabinet glovebox easier.

### Simulated saliva and lung fluid

Simulated saliva was prepared as described elsewhere (Biryukov *et al*. 2020). Simulated saliva was stored at 4°C until use and any unused portion was discarded after 1 week and a new preparation was made. Simulated lung fluid was prepared based upon the work of Hassoun *et al*. (2018) and Kumar *et al*. (2017) and modified to use Hanks’ Balanced Salt Solution (HBSS) as the diluent for the protein and antioxidant components (Bicer 2014).

Virus was concentrated and resuspended in simulated saliva or simulated lung fluid by using a centrifugal concentrator (Spin‐X UF Concentrator, Corning Cat. No. CLS431491, Corning, NY) with a 100 kiloDalton (kDa) molecular weight cut‐off that retained the SARS‐CoV‐2, but allowed the complete cell culture media components to be removed. The retentate virus was resuspended (i.e. *quantum satis*; Q.S.) with either the simulated saliva or simulated lung fluid prior to spiking representative FFR coupons or whole masks. Virus concentrations in simulated saliva were 2 to 5 x 10^5^ TCID_50_ ml^‐1^ while virus concentration in simulated lung fluid were 9 x 10^5^ TCID_50_ ml^‐1^.

### Virus inactivation assays

Inactivation was performed with representative coupons from the FFRs or with whole masks as indicated. Coupons were 2 cm x 2 cm and multiple coupons were cut from each FFR. Coupons were inoculated with 100 μL of SARS‐CoV‐2 stock by placing nine droplets of 11·1 μl each onto the coupon. Droplets were allowed to dry on the coupons by evaporation (1–2 hours). Coupons were either treated directly or put into a brown paper bag (Great Value, Walmart, Bentonville, AR) and closed with a staple as indicated. Positive controls, coupons, or whole masks were tested in triplicate and used to determine the starting titre of virus inoculated on the test samples. Treated samples, in triplicate, were subjected to moist heat for the temperature and time indicated. A negative control was included with the positive control and treated samples to confirm that material from the FFR was not affecting the viability assay. Virus was extracted from each coupon by placing it in 10 ml of complete cell culture media that included 5% FBS and then agitating for 15 min at 200 rotations per minute (RPM) on a platform orbital shaker. The solution was then removed from the tube and concentrated down to 2 ml using a centrifugal concentrator (Spin‐X UF Concentrator, Corning Cat. No. CLS431491) with a 100 kDa cutoff. The 2‐mL concentrated samples were then filter‐sterilized (Thermo‐Fisher Cat. No. 720‐1320, Waltham, MA) through a low‐binding, 0·2‐μm filter before being assayed for infectious virus by TCID_50_ assay.

When whole masks were used, a 2 cm x 2 cm square was dedicated on the mask and mapped to identify the location where the virus was inoculated. Once the appropriate exposure was completed, this mapped location was then excised and processed in the same manner as the coupons.

### Performance degradation testing

Assessment of performance degradation in whole FFRs included determination of the initial collection efficiency, inhalation resistance, change in strap elasticity and visual inspection. Collection efficiency and airflow resistance were determined using the 8130A automated filter tester (TSI Inc., Shoreview, MN) in a manner consistent with procedure number TEB‐APR‐STP‐0059 of the National Institute for Occupational Safety and Health (NIOSH) (Anon. 2019), except that only initial collection efficiency was measured, whereas the test standard usually measures collection efficiency with loading of 200 mg of salt. In addition, this test was a check of performance and not for recertification, and FFRs were not subjected to preconditioning at 38°C and 85% relative humidity prior to testing so that any changes in performance could be attributed solely to the moist heat decontamination exposure. Whole FFRs were glued onto the test plate using hot glue to ensure a seal along the edge and then placed into the tester. Whole FFRs were challenged with a salt aerosol and the tester reported the collection efficiency and inhalation resistance at 85 L min^‐1^. Strap elasticity was tested by performing a three‐step process where a 10‐inch section of the strap was stretched three times to 200%, 150% and then 200% of its length at a rate of 1 cm s^‐1^. On the final stretch the maximum stress and maximum load on the strap was recorded for comparison. Visual inspection examined the integrity of the filtration media, strap attachments, nose pads and exhalation valve (if present).

## Author Contributions

All authors listed contributed to this work and are entitled to authorship. Dr. Choi, Ms. Sunderman and Mr. Mladineo conducted work with SARS‐CoV‐2 in the BSL‐3 laboratory. Mr. Richardson, Mr. Keyes and Mr. Hofacre conducted the performance testing of the FFRs. Dr. Middleton designed the study, analysed the results and prepared the manuscript.

## Conflict of Interest

No conflict of interest declared.
